# Copper complexes in the triple-negative breast cancer landscape: advances and opportunities

**DOI:** 10.3389/fmolb.2026.1931263

**Published:** 2026-10-12

**Authors:** Paula Marynella Alves Pereira Lima, André Carlos Pereira de Matos, Gabriela da Silva Guimarães, Laryssa Aparecida Sales Barbosa, Thaise Gonçalves Araújo

**Affiliations:** 1 Laboratory of Genetics and Biotechnology, Institute of Biotechnology, Universidade Federal de Uberlândia, Patos de Minas, Minas Gerais, Brazil; 2 Laboratory of Nanobiotechnology Prof. Dr. Luiz Ricardo Goulart Filho, Institute of Biotechnology, Universidade Federal de Uberlândia, Uberlândia, Minas Gerais, Brazil

**Keywords:** breast cancer, chemotherapy, copper, treatment, triple negative

## Abstract

Triple-negative breast cancer (TNBC) is a subset of mammary tumors with heterogeneous biology, lacking estrogen receptor, progesterone receptor, and human epidermal growth factor receptor 2 (HER2). Patients generally exhibit an aggressive course, less favorable disease-specific outcomes, and a higher propensity for early recurrence, with chemotherapy remaining the primary systemic treatment eligible for all patients. In this sense, efforts are urgent to identify new effective compounds, aiming to increase disease-free and overall survival. Copper complexes have emerged as promising in oncology by regulating redox balance and modulating hallmarks such as proliferation, apoptosis, epithelial-mesenchymal transition, and angiogenesis. Furthermore, complexes containing copper tend to have low adverse effects. In TNBC, copper compounds may represent at least one component of a multi-action management strategy, being potentially effective against these tumors. The focus of this review is to provide information on TNBC biology and the role of copper in its signaling pathways, addressing the current copper complexes that have been explored to improve treatment.

## 1 Introduction

Cancer is one of the leading causes of morbidity and mortality worldwide, being a significant barrier to increasing life expectancy in the 21st century (Bray et al., 2024). Breast cancer (BC) is the most commonly diagnosed cancer among women, with approximately 2.3 million new cases in 2022 (Bray et al., 2024; IARC, 2026). Well-established screening programs have promoted early diagnosis of BC, and advances in treatment strategies have contributed to greater patient survival. However, this is not a reality across the world, since women living in countries classified as low or medium-Human Development Index have higher mortality rates (Arnold et al., 2022; Elbasheer et al., 2025).

BC is a heterogeneous disease with different molecular subtypes and outcomes (Malighetti et al., 2025). In clinical practice, the expression levels of estrogen receptor (ER), progesterone receptor (PR), human epidermal growth factor receptor 2 (HER2) and proliferation marker protein Ki-67 are considered, resulting in Luminal A (ER+ and/or PR+, HER2−, low Ki-67), Luminal B (ER+ and/or PR+, HER2− with high Ki-67 or HER2+ with any Ki-67 status), HER2-enriched (ER−, PR−, HER2+) and triple-negative (TN) (ER−, PR−, HER2−) classification (Zhang et al., 2023; Xiong et al., 2025).

Triple-negative breast cancer (TNBC) accounts for approximately 15%–20% of all breast tumors (Keskinkilic and Sacks, 2024), being more common in younger premenopausal women. In this group, patients more frequently carry mutations in Breast Cancer Genes (*BRCA*) (Zhu et al., 2023). TN tumors have an aggressive course, are highly invasive, and have a worse prognosis, with a median overall survival of approximately 12–14 months and low 5-year overall survival rates in the metastatic setting. TNBC shows a tendency toward distant recurrence, mainly affecting visceral sites such as the lungs, liver, and brain (Kesireddy et al., 2024; Piedra-Delgado et al., 2024). Moreover, TNBCs have complex genetic, transcriptional, and protein profiles (known as cancer cell-intrinsic characteristics) in addition to distinct biological characteristics related to their tumor microenvironment (TME), defined as extrinsic characteristics (Bianchini et al., 2022).

Locoregional management and systemic chemotherapy (including neoadjuvant regimen) remain the main treatment strategies (Basmadjian et al., 2023). Routinely used drugs include taxanes (paclitaxel, docetaxel, nab-paclitaxel), non-taxane microtubule inhibitors (eribulin, vinorelbine), anthracyclines (doxorubicin - DOX), antimetabolites (capecitabine, gemcitabine), and alkylating agents (cyclophosphamide, cisplatin, carboplatin), generally prescribed in combined regimens (Roskoski, 2024). However, TN tumors become progressively more resistant and intrinsically unresponsive, compromising the effectiveness of the treatment (Lobefaro et al., 2023). In this scenario, inorganic medicinal chemistry has emerged, with the development of drugs containing transition metals, such as copper (Cu) (Wang et al., 2024).

Cu is the third most abundant essential trace metal in organisms, being a catalytic cofactor in redox protein chemistry (da Silva D. A. et al., 2022). Cu levels are carefully regulated, as they can generate reactive oxygen species (ROS). Interestingly, this is a feature widely explored in oncology, since transformed cells are under constant oxidative stress, which is exacerbated by Cu, selectively inducing cell death. However, other signaling pathways are also regulated by Cu levels, including those closely related to TNBC progression (da Silva D. A. et al., 2022; Balsa et al., 2025). Antitumor pharmacological preparations (Rogalewicz and Czylkowska, 2025) and radiopharmaceuticals for diagnosis (Morgan et al., 2023) have even been developed. Herein, we will review the molecular characteristics of TNBC, the biological importance of Cu, and its role in the signaling pathways of these tumors. Finally, we present the potential of Cu complexes in the treatment of TNBC.

## 2 The triple-negative breast cancer

### 2.1 Definition and clinical aspects

TNBC is a pathological definition based on negative staining for the three standard immunohistochemical biomarkers, ER, PR, and HER2. The American Society of Clinical Oncology guidelines define ER/PR positivity as greater than or equal to 1%, and HER2 negativity is considered immunohistochemical expression of 0–1+ or lack of gene amplification (FISH, 2.0) (Zhang H. et al., 2020). TN tumors have moderate to high-grade cells and are highly proliferative. Relapses are earlier and more frequent than other subtypes, and their distribution is also correlated to race and/or country of origin (Zagami and Carey, 2022). In a study carried out between 2010 and 2019 in the United States, the incidence of TNBC was 33.8% in black women, 17.5% in Caucasian women, 14.7% in American Indians and Alaska Natives, 14.7% in Hispanic women, and 12.4% in Asian women (Du and Li, 2023). However, Sung and collaborators showed that this percentage varies among countries. In West Africa, TNBC affects 24.1% of black women, in the Caribbean, 21.2%, and in East Africa, it reaches 11.6% of BC cases. In fact, black women are genetically heterogeneous, and the prevalence of this subtype is related to the degree of African ancestry in the population (Sung et al., 2019).

BC has been increasing since the 1990s, associated with the westernization of behavior, sedentary lifestyle, inadequate diet, early menarche, and late pregnancy (Łukasiewicz et al., 2021). By 2030, 2.7 million cases and 870,000 deaths are expected annually on a global scale, with 15%–20% of cases classified as TNs (Łukasiewicz et al., 2021; Keskinkilic and Sacks, 2024). Compared to other subtypes, TNBCs have the lowest survival rate, reaching up to 40% mortality in the first 5 years (Yin et al., 2020). Again, black women have the lowest 3-year overall survival rates (79.4%), followed by Caucasians (83.1%), Hispanics (86.0%), and Asians (87.1%) (Wang et al., 2021). Distant metastases are observed in a substantial proportion of patients (approximately 46%), with recurrence rates around 25% after surgery. Mortality within 3 months after recurrence has been reported to reach up to 75% in some cohorts (Costa et al., 2021).

### 2.2 Pathways and subtypes

TNBC is not a unique disease, encompassing multiple entities behind the absence of expression of ER, PR, and HER2. A holistic characterization is necessary to understand these tumors, in which intrinsic and extrinsic features are integrated into a dynamic, coordinated ecosystem. Indeed, cancer development, progression, and treatment resistance are influenced by genetic and epigenetic changes (intrinsic), as well as by the interactions of tumor cells and their microenvironment (extrinsic) (Syrnioti et al., 2024).

Molecularly, different targets and mutations are described. *BRCA* mutations, for example, are strongly associated with TNBC and contribute to genomic instability, as *BRCA1/2* are essential for homologous recombination repair (Fujii, 2025). The *TP53* tumor suppressor gene is one of the most frequently mutated genes. In addition, approximately 30%–50% of TNBC patients present alterations in the phosphatase and tensin homolog gene (*PTEN*), which trigger phosphatidylinositol 3-kinase (PI3K) signaling (Lu et al., 2023). The PI3K/AKT/mTOR pathway is correlated to proliferation, metastasis, and vascular growth, contributing to an unfavorable prognosis (Liao et al., 2021; Lu et al., 2023; Zhou and Zhou, 2025). The proto-oncogene *MYC* is also frequently amplified or overexpressed in TNBC, driving cell proliferation, metabolic reprogramming, and therapeutic resistance (Zhou and Zhou, 2025). Furthermore, TNBCs are characterized by aberrant expression of immune checkpoint proteins, particularly programmed cell death 1 ligand (PD-L1) and its receptor PD-1, which suppresses antitumor immunity (Yang and Wang, 2025).

TNBCs overexpress receptor tyrosine kinases (RTKs), a family comprising a diverse range of cell surface receptors that transmit cellular signals involved in growth, motility, differentiation, and metabolism. They share a common protein structure with an extracellular ligand-binding domain, a transmembrane helix, and an intracellular region that includes a tyrosine kinase domain (TKD). Activation of these receptors promotes TKD autophosphorylation, followed by the recruitment of downstream signaling proteins and activation of MAPK and PI3K/AKT (Tomuleasa et al., 2024). The epidermal growth factor receptor (EGFR) is an important RTK and an independent prognostic marker, being overexpressed in more than half of TNBC cases, negatively correlated with patient survival (Nisticò et al., 2023; Jin et al., 2024).

TN tumors upregulate fibroblast growth factor receptors (FGFRs), which are RTKs that activate Ras/MAPK, PI3K/AKT, and PLCγ signaling pathways. The FGFR family comprises four highly conserved RTKs: FGFR1, FGFR2, FGFR3, and FGFR4 (Tomuleasa et al., 2024). In TNBC, FGFRs are associated with T cell exclusion from the TME, and their upregulation is also correlated with poorer overall survival (Wu et al., 2022). Vascular endothelial growth factor receptors (VEGFRs), a group of three homologous transmembrane RTKs that bind vascular endothelial growth factors (VEGFs), are relevant intrinsically, as they modulate angiogenesis and vascular permeability. VEGF-mediated angiogenesis in TNBC promotes immunosuppressive TME, and TNs exhibit higher VEGF expression compared to other BC subtypes (Liao et al., 2021; Tomuleasa et al., 2024; Zhou and Zhou, 2025).

Androgen receptor (AR), a member of the steroid receptor family, has been considered in TNBC, acting as a nuclear transcription factor that regulates gene expression associated with cell proliferation, invasion, and metastasis. AR expression is detected in approximately 30%–35% of TNBC cases and is associated with more favorable clinicopathological features, including lower tumor grade, reduced proliferative index, and improved disease-free survival. However, AR-positive tumors tend to exhibit lower sensitivity to chemotherapy and reduced rates of pathological complete response (pCR) (Sukumar et al., 2021; Maqbool et al., 2022).

Cell cycle regulators also play a relevant role in tumor biology. Cyclin-dependent kinases (CDKs) regulate the cell cycle and gene transcription by phosphorylating target proteins. For instance, the cyclin D–CDK4/6 complex phosphorylates the retinoblastoma protein (RB) in the nucleus, leading to loss of its tumor-suppressor function and, consequently, allowing cell-cycle progression and DNA replication (Alrouji et al., 2025). Increased CDK4/6 expression is associated with an unfavorable prognosis in patients with TNBC (41). Furthermore, a phase II randomized clinical trial by Tan et al. demonstrated that patients with TNBC treated with the CDK4/6 inhibitor trilaciclib in combination with chemotherapy had longer progression-free survival and overall survival than those receiving chemotherapy alone (Tan et al., 2019).

Finally, PD-L1 expression is also considered in TNBC, as it is directly associated with immune evasion (Zhou and Zhou, 2025). The interaction between PD-L1 expressed by tumor cells and PD-1 on T cells inhibits the proliferation of tumor-infiltrating lymphocytes (TILs) and cytokine production, thereby promoting tumor cell survival (Zhu et al., 2020).

The above intrinsic characteristics were initially analyzed at the transcriptional level, and the molecular signature of TNBC was determined by the expression of 50 genes, established as the PAM50 subtype predictor. Interestingly, this approach revealed a substantial overlap between TNBC and basal-like (BL) tumors: approximately 50%–75% of TNBC cases exhibit a basal phenotype, while about 80% of BL tumors are TN (Garrido-Castro et al., 2019). Basal epithelial cell markers include keratin 5, keratin 17, keratin 14, integrin-β4, and laminin. Mutations in *BRCA1* are present in the ER− BL phenotype. Overexpression of EGFR, amplification of *MYC*, and mutations in *TP53* are described (Verma et al., 2022; Faraldo et al., 2024). The claudin-low subtype is also TN in approximately 7 a 14% of cases and is characterized by reduced expression of cell adhesion proteins and overexpression of CD14 and CD79a. In Claudin-low tumors, CD44, vimentin, and N-cadherin are upregulated, contributing to a mammary stem cell phenotype (Pan et al., 2023).

Based on the molecular profile, Lehmann and collaborators identified six subtypes of TNBC: basal-Like 1 (BL1), basal-Like 2 (BL2), immunomodulatory (IM), mesenchymal (MES), mesenchymal stem-like (MSL), and luminal androgen receptor (LAR) (Lehmann et al., 2011). However, subsequent works demonstrated that two of the six proposed subtypes, IM and MSL, are not independent cancer cell intrinsic subtypes, but rather enriched with high numbers of TILs or mesenchymal stem cells (MSCs), respectively (Lehmann et al., 2016). In this way, the molecular subtypes of TNBC were refined into BL1, BL2, MES, and LAR (Figure 1).

**FIGURE 1:**
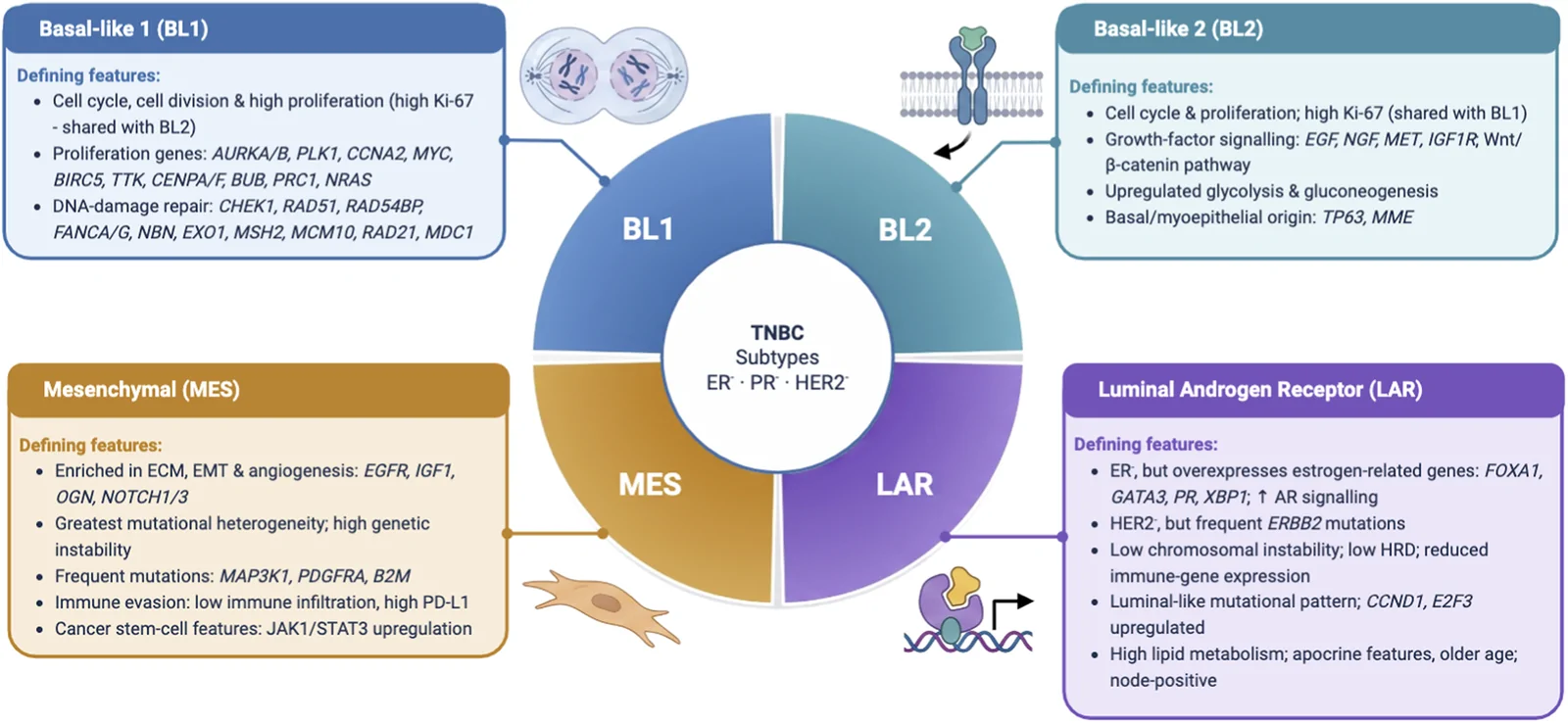
Main characteristics of the different subtypes of triple-negative breast cancer: Basal-like 1 (BL1), Basal-like 2 (BL2), Mesenchymal (MES), and Luminal androgen receptor (LAR). Data are aggregated from Lehmann et al. (Lehmann et al., 2016). ECM: extracellular matrix; EMT: epithelial-mesenchymal transition; AR: androgen receptor; HRD: homologous recombination deficiency; PD-L1: programmed death-ligand 1; ER: estrogen receptor; PR: progesterone receptor; HER2: human epidermal growth factor receptor 2. The figure was created with BioRender.com. Barbosa, L. (2026) https://BioRender.com/iiu3osv.

BL1 and BL2 are enriched with gene ontologies of the cell cycle and cell division components and pathways, expressing genes associated with proliferation such as aurora kinase A (*AURKA*), aurora kinase B (*AURKB*), centromere protein A (*CENPA*), centromere protein F (*CENPF*), mitotic checkpoint protein bub (*BUB*), TTK protein kinase (*TTK*), cyclin A2 (*CCNA2),* protein regulator of cytokinesis 1 (*PRC1*), MYC, proto-oncogene NRAS (*NRAS*), polo like kinase 1 (*PLK1*) and baculoviral IAP repeat containing 5 (*BIRC5*). They also have high levels of Ki-67. In BL1, this signature is accompanied by the expression of genes associated with the repair system, such as checkpoint kinase 1 (*CHEK1*), FA complementation group A (*FANCA*), complementation group G (*FANCG*), DNA-dependent ATPase RAD54 (*RAD54BP*), RAD51 recombinase (*RAD51*), nibrin (*NBN*), exonuclease 1 (*EXO1*), MutS homolog 2 (*MSH2*), minichromosome maintenance 10 replication initiation factor (*MCM10*), RAD21 cohesin complex component (*RAD21*) and mediator of DNA damage checkpoint 1 (*MDC1).* The BL2 subtype expresses genes related to growth factor signaling pathways such as *EGF*, nerve growth factor (*NGF*), MET proto-oncogene (*MET*), insulin-like growth factor 1 receptor (*IGF1R*), and genes associated with the wingless (Wnt)/β-catenin pathway. Genes related to glycolysis and gluconeogenesis are upregulated. The BL2 subtype exhibits characteristics suggestive of a basal/myoepithelial origin, as evidenced by the expression of the tumor protein p63 (*TP63*) and membrane metalloendopeptidase (*MME*) genes (Lehmann et al., 2011; Lehmann et al., 2016; Lehmann et al., 2021).

The MES subtype, in turn, is enriched in pathways related to the extracellular matrix (ECM), epithelial-mesenchymal transition (EMT), and angiogenesis, expressing *EGFR*, *IGF1*, osteocyte markers (*OGN*), *NOTCH1*, and *NOTCH3* (Bareche et al., 2018; Jiang et al., 2019; Lehmann et al., 2021). This subtype is marked by the greatest heterogeneity of mutations among TN, with higher genetic instability. The MES subtype exhibits immune evasion mechanisms, characterized by low immune cell infiltration and high PD-L1 expression. Mutations in mitogen-activated protein kinase kinase 1 (*MAP3K1*), platelet-derived growth factor receptor alpha (*PDGFR-A*), and beta-2-microglobulin (*B2M*) are frequent (Bareche et al., 2018; Lehmann et al., 2021). Furthermore, the MES gene expression pattern has characteristics of breast cancer stem cells (BCSCs) and upregulation in JAK1/STAT3 signaling (Jiang et al., 2019).

Finally, LAR subtype is ER−, but overexpresses estrogen-related genes, such as forkhead box A1 (*FOXA1)*, GATA binding protein 3 (*GATA3*)*, PR* e X-box binding protein 1 (*XBP1*), with increased AR signaling. Although clinically classified as HER2−, they exhibit more frequent mutations in the Erb-B2 receptor tyrosine kinase 2 (*ERBB2*) compared to other TN subtypes. The LAR subtype has low chromosomal instability, reduced expression of immune-related genes, a low homologous recombination deficiency (HRD) score, and mutational patterns similar to those observed in luminal tumors. They are associated with high lipid metabolism, advanced age, positive lymph nodes, and apocrine characteristics (Asleh et al., 2022). The cyclin D1 (*CCND1*) and E2F transcription factor 3 (*E2F3*) genes are upregulated (Jiang et al., 2019).

Beyond intrinsic tumor characteristics, extrinsic factors related to TME should also be considered in the context of TN. The TME is a highly coordinated ecosystem composed of cancer cells surrounded by non-malignant cells, jointly embedded in an altered, vascularized ECM. Myeloid cells, cancer-associated fibroblasts (CAFs), mesenchymal stem cells (MSCs), and adipocytes, as well as other cell types, are present, regulating proliferation, angiogenesis, apoptosis, immunosuppression, and drug resistance (Deepak et al., 2020; de Visser and Joyce, 2023; Guo and Xu, 2024).

The ECM is an acellular three-dimensional structure in which its components constantly interact with cells, transmitting signals that regulate adhesion, migration, proliferation, apoptosis, survival, and differentiation (Deepak et al., 2020; Wang et al., 2025b). In the TN subtype, there is a significant enrichment of collagen deposition and ECM stiffness when compared to luminal tumors. This phenomenon results from an increase in the number of fibroblasts, which drive matrix remodeling (Acerbi et al., 2015; Park et al., 2015). In TNBC, the main biomarkers of EMT are N-cadherin, TGF-β, vimentin, matrix metalloproteinases (MMP), heparanases, lysyl oxidase (LOX), and proteoglycans/glycosaminoglycans (Zolota et al., 2021).

Myeloid cells are involved in multiple stages of tumor-promoting processes, including angiogenesis, immunosuppression, and progression. Immunosuppressive myeloid cells may be induced by interleukin-34 (IL-34), associated with chemoresistance. IL-34 is overexpressed in TNBC compared to other subtypes and is considered a poor prognostic factor (Kajihara et al., 2023). In addition, the IL-6/STAT3 signaling pathway modulates TME, triggering immune evasion mediated by CAFs (Morrow et al., 2023). CAFs, in turn, are one of the most frequent stromal cells and contribute significantly to the immunosuppressive TME. Tumor-associated macrophages (TAMs), together with CAFs, hinder antigen presentation and inhibit adaptive immunity, leading to immune resistance in TNBC (Xu J. et al., 2024). Furthermore, they secrete important cytokines such as transforming growth factor beta (TGF-β1), platelet-derived growth factor (PDGF), and IL-6, inducing metastases (Hill et al., 2020).

TNBC is highly immunogenic, and high levels of TILs have been correlated with better short- and long-term prognosis and a better response to neoadjuvant chemotherapy (Binnewies et al., 2018). High levels of TILs are associated with a larger population of natural killer (NK) cells and an enhanced capacity to identify tumor neoantigens, thereby boosting the adaptive immune response. However, the impact of these cells varies according to TNBC subclassification (Huertas-Caro et al., 2023; Han et al., 2024). In parallel, adipocytes involved in BC progression are activated in the TME of TNBC, being structurally different from those residing in normal adipose tissues. In tumors, they are smaller, with lower lipid content, and capable of promoting immunosuppressive TME (Liu et al., 2021). Finally, MSCs have peculiar characteristics, such as self-renewal and the ability to differentiate into different cell types (Hill et al., 2020). MSCs recruited to the TME originate mainly from bone marrow and adipose tissue and release factors that contribute to therapy resistance and BC progression (Augimeri et al., 2023).

Tumor cells communicate with the ECM and can induce EMT (Fujisaki and Futaki, 2022). In this event, a phenotypic change promotes the migration of transformed cells, promoting metastasis, which is frequent in TNBC. Indeed, TNBC cells are often enriched with mesenchymal features (Chen and Zhao, 2025). TGF-β is a key inducer of EMT, activating canonical (SMAD-dependent) and non-canonical signaling pathways, including PI3K/AKT/mTOR, RAS/RAF/MEK/ERK, and Wnt/β-catenin (Poursani et al., 2023). Therefore, intrinsic characteristics regulate TME and influence the aggressiveness of TNBC (Xiao et al., 2019). In this scenario, a better understanding of cellular heterogeneity within the TME of TNBC can reveal predictive biomarkers and improve existing treatments, including the development of new compounds.

### 2.3 Treatment

TNBC has been one of the major focuses of translational research, and different efforts have been adopted to control this tumor (Figure 2). Despite recent advances, conventional and potentially toxic therapies remain the mainstay of treatment. In early-stage disease, the gold-standard treatment approach is adjuvant chemotherapy based on anthracyclines and taxanes. The introduction of platinum salts in combination with standard neoadjuvant chemotherapy has substantially improved pCR in patients with stage II-III TNBC. However, current clinical guidelines do not support the universal use of this regimen due to the absence of definitive survival benefits (Wu et al., 2025). Patients with residual disease after neoadjuvant chemotherapy are usually treated with postoperative capecitabine to prolong survival (Capuozzo et al., 2023). In this context, therapeutic agents targeting pathways relevant to TNBC have been widely investigated. Among these approaches, inhibitors targeting the PI3K/AKT/mTOR pathway (including ipatasertib and capivasertib), AR antagonists (such as enzalutamide), and FGFR and VEGFR inhibitors (futibatinib and ramucirumab) have been promising in selected patient subgroups. However, many of these agents are still under clinical investigation, with variable efficacy depending on the tumor’s molecular profile (Vagia et al., 2020; Jie et al., 2025).

**FIGURE 2:**
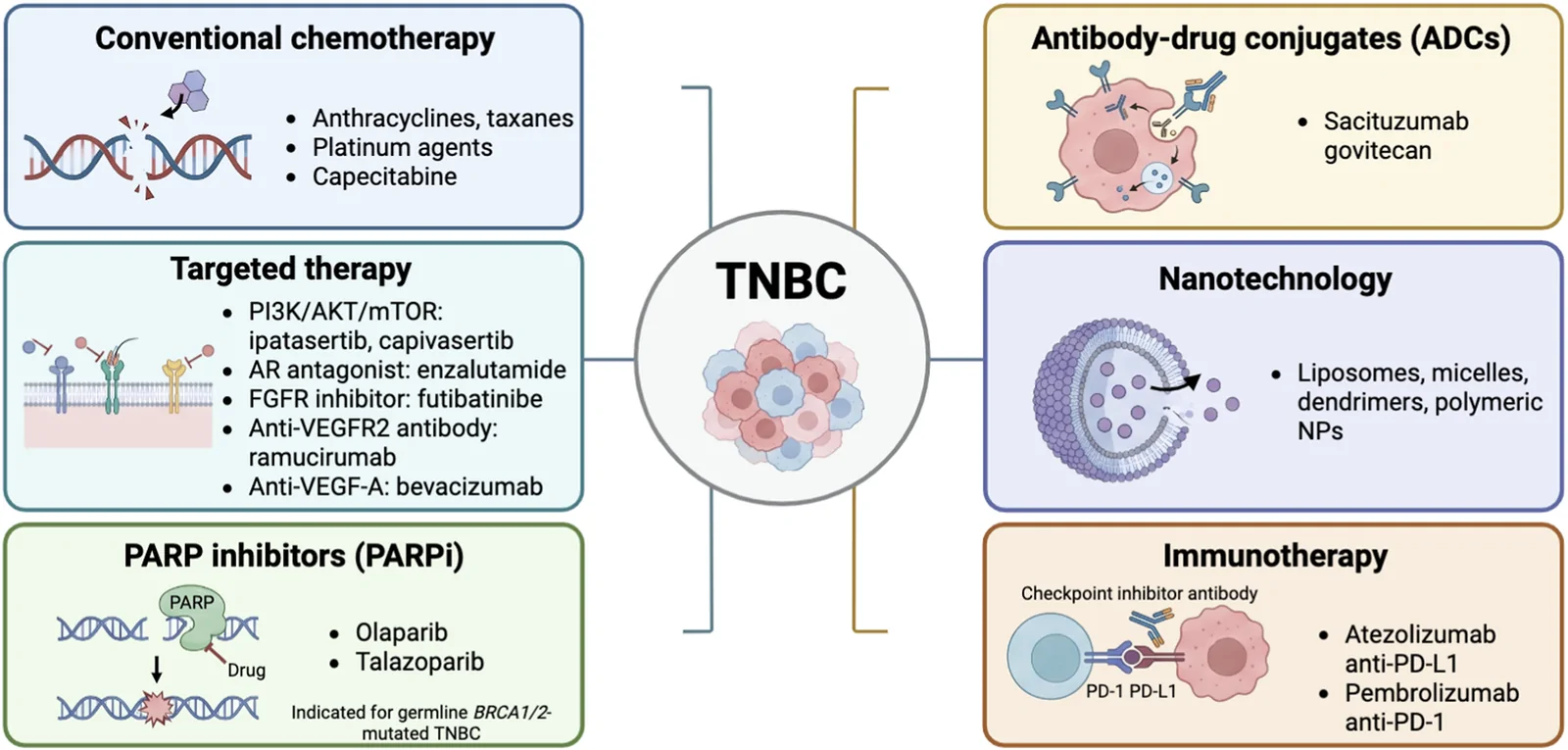
Therapeutic strategies adopted for triple-negative breast cancer: conventional chemotherapy, inhibitors targeting canonical signaling pathways, poly (ADP-ribose) polymerase inhibitors (PARPi), antibody-drug conjugates, nanotechnology, and immunotherapy. CT: chemotherapy; pCR: pathological complete response; AR: androgen receptor; FGFR: fibroblast growth factor receptors; VEGFR: vascular endothelial growth factor receptor; TNBC: triple negative breast cancer, BRCA: breast Cancer Genes; TROP2: trophoblast surface antigen 2; TOP 1: topoisomerase I; NPs: polymeric nanoparticles; PD-L1: programmed cell death 1 ligand; FDA: Food and Drug Administration. The figure was created with BioRender.com. Barbosa, L. (2026). https://BioRender.com/sjcul9u.

Poly (ADP-ribose) polymerase inhibitors (PARPi), such as olaparib and talazoparib, have been indicated for patients with *BRCA* mutations. A Phase III clinical trial involving patients with *BRCA*-mutated TNBC treated with olaparib demonstrated greater 3-year invasive disease-free survival in the treatment group (87.5%) compared to the placebo group (80.4%), with fewer deaths in the group that received olaparib (Tutt et al., 2021). Although currently approved PARPi have demonstrated significant clinical benefits, their toxicity and resistance continue to limit their use (Ray and Opyrchal, 2025).

Bevacizumab, a recombinant humanized monoclonal antibody targeting circulating VEGF-A, impairs tumor neovascularization. Clinical studies have demonstrated an increase in pCR rates in the neoadjuvant setting; however, its use has also been associated with higher rates of adverse events, such as hypertension, neutropenia, and mucositis, which may lead to dose reduction and postoperative complications (Vagia et al., 2020). Antibodies have also been used as immunotherapeutics. In 2019, atezolizumab was approved by the Food and Drug Administration (FDA) for the first-line treatment of PD-L1-positive TNBC. The IMpassion130 trial demonstrated clinical benefit with the combination of atezolizumab and nab-paclitaxel, including improved progression-free survival, particularly in PD-L1-positive patients. However, no significant overall survival was observed in the overall population (Narayan et al., 2020; Xu et al., 2025).

Antibody–drug conjugates (ADCs) are emerging strategies in which recombinant monoclonal antibodies are covalently bound to cytotoxic agents via synthetic linkers. In 2020, the FDA approved sacituzumab govitecan (SG) for TN tumors. SG is a humanized monoclonal antibody targeting trophoblast surface antigen 2 (Trop-2) conjugated to topoisomerase I (TOP1) inhibitor (Popovic et al., 2023; Caputo et al., 2024). In the phase III ASCENT trial, in patients without brain metastases, SG demonstrated significant clinical benefit, with a median progression-free survival of 5.6 months and a median overall survival of 12.1 months compared to 6.7 months in the control group (Bardia et al., 2021). Based on these findings, SG received further FDA approval in 2021 for patients with locally advanced or metastatic TNBC who had received at least two prior systemic therapies (Carey et al., 2022).

Nanotechnology has also been explored for the treatment of TNBC, with the development of nanoformulations and optimization of their surface charge, size, and conformation for targeted drug delivery. Liposomes, polymer conjugates, micelles, polymeric nanoparticles (NPs), dendrimers and carbon-based formulations are used, with improved delivery and reduced resistance (Pradhan et al., 2023). For TNBC, liposomes carrying DOX and rapamycin, siRNA-conjugated poly (amidoamine) dendrimers for depletion of the TWIST transcription factor, and the agents Gd-DOTA dendrimers for imaging and drug delivery are described. L-lactic-co-glycolic acid in polymeric nanoparticles showed significant tumor inhibition in TNBC mouse models (Singh and Yadav, 2021).

Among the events modulated by the different treatment strategies adopted, oxidative stress deserves special attention. ROS play essential roles in normal cellular physiology and are produced through both enzymatic and non-enzymatic pathways. ROS can be generated through multiple cellular mechanisms, with the mitochondrial respiratory chain serving as a primary intracellular source, including superoxide anion (O_2_•−), which, in the cytoplasm, can be rapidly converted into hydrogen peroxide (H_2_O_2_). H_2_O_2_ also contributes to the generation of hydroxyl radicals (•OH) via the Fenton reaction. During normal metabolism, the synthesis of adenosine triphosphate (ATP) produces ROS. NAPH oxidases (NOX) transfer electrons across biological membranes and generate O_2_•−, which is subsequently converted into H_2_O_2_ through the activity of extracellular superoxide dismutase (SOD3). Mitochondrial complexes produce O_2_•−, which is metabolized to H_2_O_2_ by manganese superoxide dismutase (SOD2) in the mitochondrial matrix or by SOD1 in the intermembrane space. On the other hand, the Fenton pathway generates •OH radical through the decomposition of H_2_O_2,_ independently of enzymatic activity. •OH is highly reactive and can interact with proteins, carbohydrates, lipids, and nucleic acids (Cheung and Vousden, 2022).

Although important, excessive ROS is associated with several diseases, including cancer. Oxidative stress can induce damage to lipids, proteins, and DNA, leading to deletions, chromosomal rearrangements, and mutations in tumor suppressor genes and proto-oncogenes. In addition, ROS can activate signaling pathways associated with tumor progression, such as PI3K/AKT/mTOR (Bel’skaya and Dyachenko, 2024). In this context, ROS and redox adaptation play central roles in the biology of TNBC (Cruz-Gregorio, 2026). DOX, for example, is a first-line drug for the treatment of patients with TNBC. The metabolic conversion of DOX increases ROS production, promoting lipid peroxidation, mitochondrial instability, and, consequently, cell death. The use of DOX is limited due to its toxicity (Srinivas et al., 2019; Lee et al., 2021).

Interestingly, ROS-mediated cytotoxicity is merely one effect associated with redox modulation. At moderate levels, reflecting the chronic, adaptive redox state of a growing tumor, ROS act as pro-tumorigenic signaling molecules, regulating oncogenes and tumor suppressor genes. ROS promote proliferation and extracellular matrix remodeling, sustain invasion and angiogenesis, and modulate anti-tumor immunity (Attique et al., 2025). Al Haq et al. (2022) demonstrated that, in TNBC, there is pro-oxidant production of mitochondrial ROS that supports BCSCs, IL-6 secretion, and macrophage polarization toward an immunosuppressive M2 phenotype. Thus, in TNBC, ROS act not only as an outcome of cytotoxic therapy but also as upstream signaling intermediates that sustain stemness, immune evasion, and metastatic competence. This duality underscores the need to consider the ROS threshold, subcellular source, and duration of exposure, rather than ROS levels in isolation, when developing therapies involving metabolic alteration.

Depletion of mitochondrial Cu increases oxidative stress and impairs mitochondrial function, leading to apoptosis. This strategy has been effective against tumors that depend on oxidative phosphorylation, such as TNBC (Cui et al., 2021). Furthermore, cells can die by a Cu-dependent mechanism called cuproptosis. Cuproptosis is a mechanism involving lipoic acid-mediated protein lipoylation. Lipoic acid acts as a direct Cu ligand, leading to the aggregation of lipoylated proteins, the loss of Fe-S cluster proteins, and increased levels of heat shock protein 70 (HSP70) in response to acute proteotoxic stress. Furthermore, ferredoxin 1 (FDX1) reduces Cu(II) to Cu(I), thereby activating the lipoylation machinery and Cu toxicity. Consequently, cells dependent on mitochondrial respiration are approximately 1,000 times more sensitive to copper ionophores than cells relying on glycolysis. (Tsvetkov et al., 2022). Sun et al. (2025) demonstrated that Cu overload in TNBC paradoxically activates AKT1 signaling, which phosphorylates FDX1, thereby compromising its activity. This AKT1–FDX1 axis not only attenuates cuproptosis but also redirects cellular metabolism toward the glycolytic pathway (Sun et al., 2025). A large-scale drug repurposing screen identified the FDA-approved compound zinc pyrithione as a cuproptosis inducer capable of inhibiting TNBC progression, reinforcing that the pharmacological reactivation of the FDX1-dependent pathway is a viable therapeutic strategy (Yang et al., 2023). Molecularly, patients with TNBC were previously evaluated for the expression of cuproptosis-related genes (CRGs) and their correlation with immune cells, clinicopathological characteristics, and prognosis. Lower CRG scores, called an immune-inflamed phenotype, were observed in samples with greater immune cell infiltration, associated with improved survival outcomes. In contrast, high CRG scores, called an immune-desert phenotype, were associated with activation of stromal pathways and immunosuppression (Sha et al., 2022). These data highlight the role of Cu in the pathological myriad of TNBC, as a cuproptosis-related metal, which needs further investigation.

## 3 Biological importance of Cu

Cu, after zinc and iron, is the third most abundant essential trace metal in human beings, with redox activity and the ability to donate and receive electrons, alternating between the reduced state Cu(I) and the oxidized state Cu(II) (da Silva D. A. et al., 2022). This property underlies the essential biological role of Cu in redox reactions, serving as a catalytic cofactor for numerous Cu-dependent enzymes, such as cytochrome c oxidase (COX), superoxide dismutase (SOD), tyrosinase, lysyl oxidase (LOX), and amine oxidases. (Genchi et al., 2025). Therefore, cells maintain an efficient distribution of Cu, ensuring its homeostatic levels. However, if Cu stores are deregulated, oxidative stress and cytotoxicity may occur. In this context, Cu produces free radicals through the Fenton principle, similar to what occurs with iron (Yang et al., 2024; Genchi et al., 2025).

Cu is an essential micronutrient absorbed in the gastrointestinal tract from foods such as liver, fish, crustaceans, nuts, and vegetables. 1n humans, total body Cu content ranges from 50 to 120 mg, and its systemic levels are tightly regulated (Genchi et al., 2025). Intracellular Cu concentration is controlled by a complex network of metal-dependent proteins, such as cuproenzymes, chaperones, and membrane transporters. These proteins work together in the import, export, and intracellular use of Cu, maintaining its homeostatic levels (Yang et al., 2024; Genchi et al., 2025; Liu et al., 2026). Dietary Cu is primarily absorbed in the duodenum and small intestine. Before uptake, Cu(II) is reduced to Cu(I) by metalloreductases, including six-transmembrane epithelial antigen of the prostate (STEAP) proteins and duodenal cytochrome B (DCYTB). Cu uptake by intestinal epithelial cells is mainly mediated by the high-affinity copper transporter 1 (CTR1), located on the apical membrane of enterocytes. After absorption, Cu(I) is released into the bloodstream, where it binds to soluble carriers such as albumin and transcuprein. Upon reaching the liver, hepatocytes take up Cu(I) via CTR1. Intracellularly, Cu(I) is distributed by copper chaperones to specific target proteins or sequestered by metallothioneins for storage. CTR1, encoded by the solute carrier family 31 member 1 (*SLC31A1*) gene, plays a critical role in cellular Cu(I) uptake across different tissues (Chen et al., 2022; Liu et al., 2026). Figure 3 summarizes the Cu metabolism.

**FIGURE 3:**
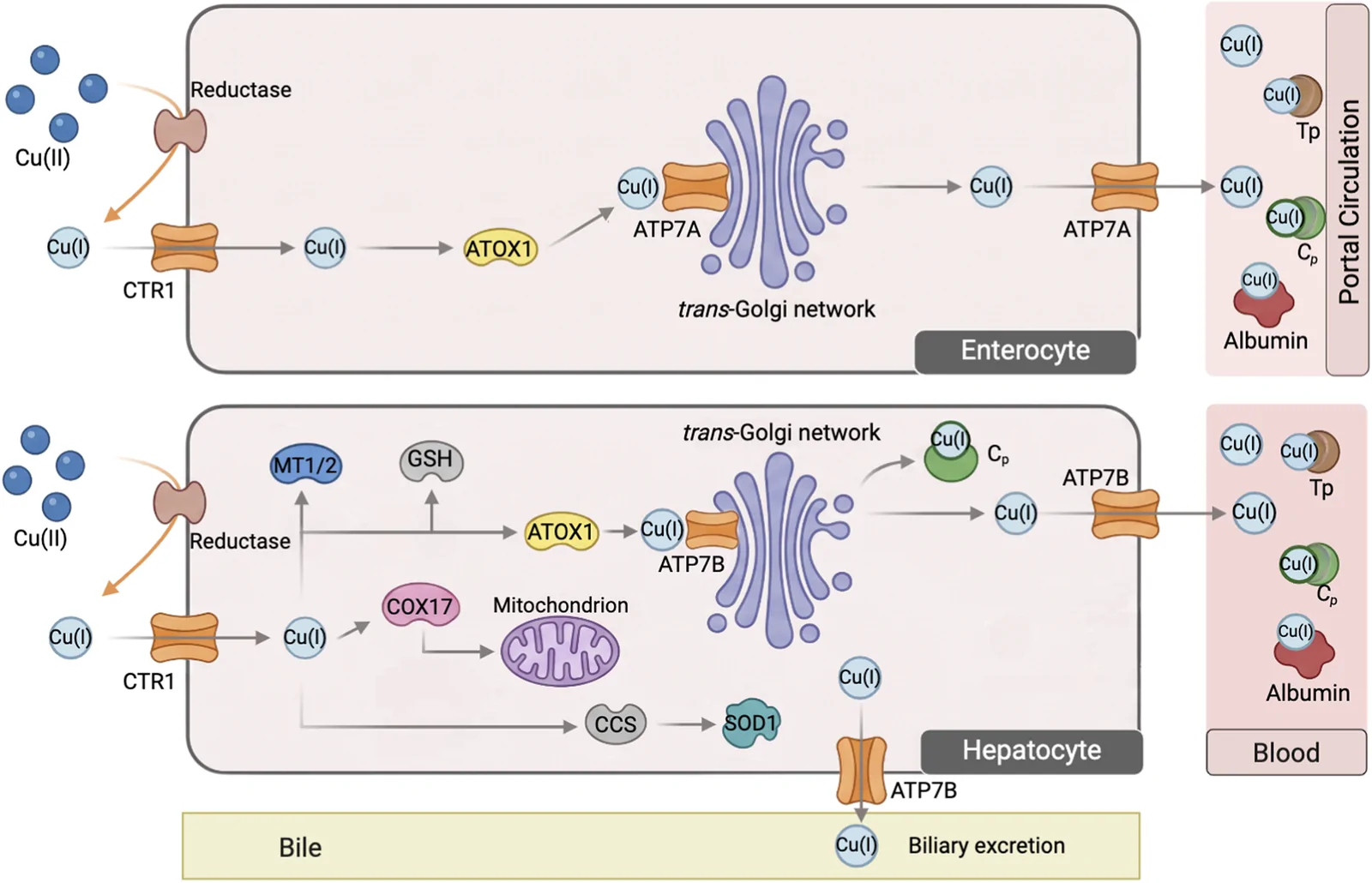
Schematic illustration of copper (Cu) metabolism at the molecular and cellular levels in mammals. Reduced Cu ions—Cu(I)—are transported by the high-affinity copper transporter protein (CTR1) at the apical surface of enterocytes. When administered via other routes, such as intraperitoneally, C(I)u also enters hepatocytes via CTR1. Copper-transporting ATPase alpha (ATP7A) translocates to the basolateral membrane to pump Cu(I) into the blood; intracellularly, Cu(I) is distributed by the antioxidant-1 chaperone (ATOX1) and directed to copper-zinc superoxide dismutase (SOD1) by the copper chaperone for superoxide dismutase (CCS). Excess Cu(I) is sequestered by metallothioneins 1 and 2 (MT1 and MT2). Elevated Cu(I) levels are also transported by ATP7B for biliary excretion. The cytochrome c oxidase chaperone (COX17) directs Cu(I) to mitochondrial Cu-dependent enzymes. ATP7B in the trans-Golgi network (TGN) also directs Cu(I) to organelles or out of the cell. In the portal circulation, Cu(I) is transported via albumin, ceruloplasmin (Cp), and transcuprein (Tp). The figure was created with BioRender.com. Barbosa, L. (2026). https://BioRender.com/d5pggel.

Intracellularly, Cu(I) trafficking is finely regulated by a network of high-affinity transporters. The chaperone antioxidant-1 (ATOX1) transfers Cu(I) to ATP7A and ATPase copper transporting beta (ATP7B) in the trans-Golgi network, facilitating the synthesis of cuproenzymes such as LOX, tyrosinase, and ceruloplasmin. Cu(I) is transported to mitochondria by COX17 and COX11, contributing to oxidative phosphorylation and mitochondrial function. In addition, Cu(I) is delivered to cytosolic proteins by copper chaperone for superoxide dismutase (CCS), which transfers Cu(I) to SOD1 (Chen et al., 2022).

ATP7A and ATP7B are responsible for Cu(I) efflux into the extracellular environment. These proteins translocate from the trans-Golgi network to vesicular compartments and fuse with the plasma membrane, releasing excess intracellular Cu(I) (Zhou and Zhang, 2023). Metallothioneins 1 and 2 (MT1 and MT2) are cysteine-rich proteins with high affinity for binding multiple Cu(I) ions. These proteins regulate Cu(I) storage, distribution, and detoxification. MT1 and MT2 also contribute to the delivery of Cu(I) to Cu-dependent enzymes, which are transcriptionally induced in response to elevated metal concentrations. Due to their high cysteine content, MTs form stable Cu–thiolate complexes, protecting cells from Cu-induced toxicity (Babak and Ahn, 2021). Therefore, the cellular balance of bioavailable Cu is highly regulated, with cuproplasia being Cu-dependent cell growth and proliferation. Cu has significant implications in tumor progression and cellular processes such as angiogenesis, metastasis, and immune evasion. Cu can also mediate cell death through cytotoxicity triggered by increased mitochondrial-dependent energy metabolism and ROS accumulation (Ge et al., 2022).

Cu homeostasis is maintained within a narrow range by ATP7A and ATP7B. Their deficiency compromises essential cuproenzymes such as lysyl oxidase and dopamine β-hydroxylase (Menkes disease) and promotes hepatic and cerebral copper accumulation (Wilson disease) (Więcek and Paprocka, 2024). Indeed, Cu deficiency predominantly impairs the function of cuproenzymes associated with, for example, cytochrome c oxidase-dependent mitochondrial respiration and SOD1-mediated antioxidant defense. Excess Cu, in turn, promotes direct structural damage through ROS generation via the Fenton reaction and mitochondrial proteotoxic stress with the aggregation of lipoylated proteins and activation of cuproptosis (Tsvetkov et al., 2022). In the oncological context, both scenarios, excess and depletion of Cu, are described. However, identifying specific tumor subtypes and profiles is important for a more precise response (Lu et al., 2025).

### 3.1 Role of Cu in TNBC signaling pathways

In TNBC cells, mitochondria have an increased Cu requirement and a metabolic preference to import and utilize lipids, instead of glucose, as an energy source (Cui et al., 2021). Thus, when Cu is depleted, the synthesis and function of COX in the electron transport chain are compromised, altering mitochondrial metabolism (Cui et al., 2021; Hao et al., 2026). Under these conditions, TNBC cells shift toward glycolysis as a compensatory metabolic strategy to maintain cellular energy demands, consequently generating less ATP than oxidative phosphorylation. Endogenous antioxidants are downregulated, and oxidative stress is increased. As redox balance is intrinsically related to cellular Cu availability, SOD activity is also compromised in the absence of this metal. Additionally, Cu depletion promotes overexpression of AMP-activated protein kinase (AMPK) in TNBC cells, with a reduction in mitochondrial ATP synthesis (oxidative), compared to glycolytic ATP (Hao et al., 2026).

Cu is also an important catalytic cofactor of enzymes that remodel the ECM. LOX is a Cu-dependent amine oxidase that promotes cross-linking of collagen and elastin (Shanbhag et al., 2019; Li et al., 2024). Collagen is an important component of the ECM and, therefore, LOX activity directly contributes to its organization and stability. Moreover, LOX family proteins are strongly associated with malignant transformation, migration, adhesion, and invasion of TNBC cells, participating in the formation of a pre-metastatic microenvironment (Li et al., 2024).

TNBC cells, which typically exhibit a higher basal Cu demand to sustain proliferation, angiogenesis, and accelerated mitochondrial metabolism, simultaneously become more vulnerable to depletion and more susceptible to exceeding the toxic threshold of Cu levels. Cu depletion modulates EMT by inhibiting canonical and non-canonical TGF-β signaling. Moreover, the reduction of intracellular Cu alters the expression of EMT-associated genes, including *TGFB2*, *MMP-2*, *MMP-9*, *MMP-14*, E-cadherin (*CDH1*), vimentin (*VIM*), zyxin (*ZYX*), and versican (*VCAN*), as well as the transcription factors SNAI1/Snail, SNAI2/Slug, ZEB1, and SPARC (Poursani et al., 2023). On the other hand, excess or redistributed intracellular Cu overwhelms the antioxidant capacity of cancer cells, making them susceptible to ROS-mediated cytotoxicity that triggers apoptosis and, when a threshold of mitochondrial proteotoxic stress is reached, cuproptosis (Sun et al., 2025). Together, these findings illustrate that Cu depletion and Cu overload act on largely non-overlapping molecular circuits in TNBC.

## 4 Cu complexes in oncology

The discovery of the anticancer properties of cisplatin (PtCl_2_(NH_3_)_2_) by Barnett Rosenberg in the 1960s was a milestone in the history of metal-based compounds and their relationship with oncology (Rosenberg et al., 1967; Rosenberg et al., 1969). Currently, cisplatin and its analogues are antitumor drugs used in 50% of cancer treatments, including ovarian, lung, breast, and colorectal cancer. However, the drug is toxic and faces resistance mechanisms and side effects (Alvarez et al., 2022; Zhang et al., 2022; Gomes et al., 2024). Despite these limitations, interest in metals remains, as they display unique characteristics, including redox potential, variable coordination models, and reactivity with organic substrates. These properties are important in the design of metal complexes that selectively bind to biomolecules and modulate different signaling pathways (Gomes et al., 2024). In this context, essential metals have been less toxic than platinum, gold, or silver (Lucaciu et al., 2022).

The medicinal value of Cu has been recognized for centuries. The Egyptians were the first to mention the antimicrobial effects of Cu, using containers made of this metal to purify water and treat wounds. Hippocrates used Cu compounds to treat wounds and ulcers (Arendsen et al., 2019). Currently, Cu complexes have been explored in oncology (Machado et al., 2021). In fact, the altered metabolism of cancer cells and the differential response between normal and transformed cells to Cu paved the way for medicinal chemistry centered on this metal (Kong and Sun, 2023). Furthermore, the combination of Cu ligands in the development of complexes modulates the targeting and cytotoxic properties. Sulfur and phosphorus donor ligands, for example, stabilize Cu(I), while nitrogen and oxygen donor ligands preferentially stabilize Cu(II). The ligand also modulates the lipophilic and hydrophilic characteristics of the resulting complexes, thereby influencing their solubility in extracellular fluids and their ability to permeate the lipid bilayer (Tardito and Marchiò, 2009). However, a systematic analysis is required to validate the activity of the complex, or rather, the role of Cu in the observed effects (Hangan et al., 2023).

Studies on Cu-based metal complexes have significantly expanded over the past decades (Zehra et al., 2021). Cu-complexes have been well tolerated (Adhikari et al., 2024), and those linked to phenanthroline have been evaluated, including against tumor cell lines with resistance to cisplatin (Ng et al., 2014; Machado et al., 2021). The mechanisms of action of Cu-based complexes (Figure 4) are unique and include inhibition of TOP1 and TOP2 (Molinaro et al., 2020), generation of ROS with degradation of genomic DNA, lipid peroxidation, cell cycle arrest in G0/G1 and apoptosis (Machado et al., 2021), control of angiogenesis, proteasome inhibition, and telomerase blockade (Zehra et al., 2021).

**FIGURE 4:**
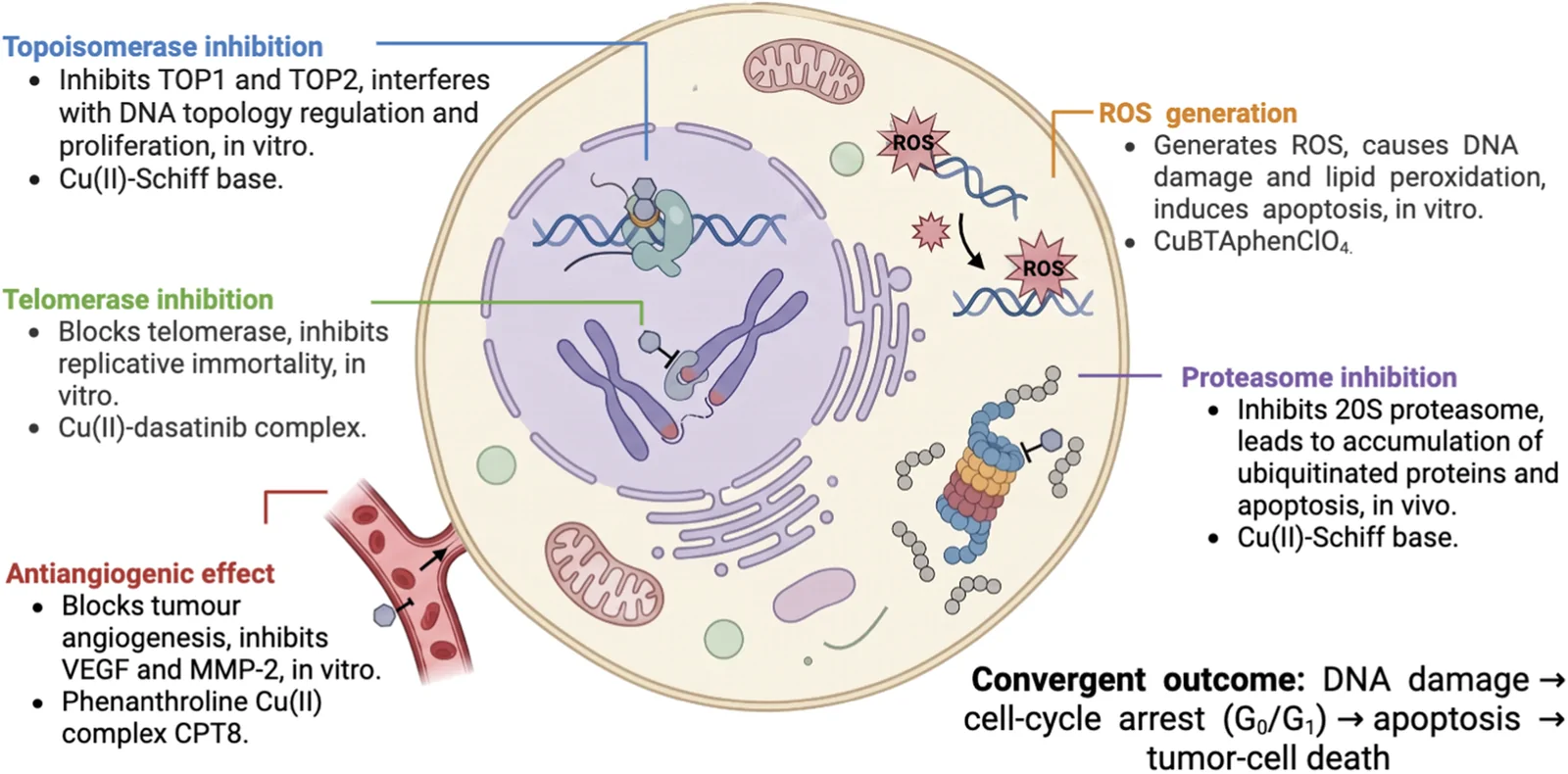
The main mechanisms of copper complexes as antitumor agents include topoisomerase inhibition, reactive oxygen species (ROS) generation, antiangiogenic effects, and inhibition of the proteasome and telomerase. The figure was created with BioRender.com. Barbosa, L. (2026). https://BioRender.com/biznhvh.

### 4.1 Inhibition of topoisomerases

Topoisomerases are enzymes that control the three-dimensional structure of DNA through coiling, linearization, and supercoiling during replication and transcription. Class I enzymes (TOP1) break the single strand of the double helix, relaxing it. Class II (TOP2) enzymes induce cuts in both ribbons to unwind them. Drugs that exploit these characteristics for therapeutic purposes form a drug-enzyme-DNA complex, preventing the proliferation of tumor cells (Shen et al., 2025). Topkaya and co-workers showed that a Cu(II) complex bound to the perchlorate ion containing a Schiff base ligand (C_19_H_17_Cl_3_CuN_4_O_6_) inhibited the activity of TOP1 and TOP2 (Gökçe Topkaya et al., 2022).

### 4.2 ROS scavengers

Cu complexes generate ROS, which is one of the main mechanisms explored. Many studies focus on the relationship between oxidative stress and apoptosis. ROS damage DNA, triggering pro-apoptotic signals when the damage cannot be repaired (Li et al., 2026). Polloni and colleagues showed that a Cu(II) complex, CuBTAPhenClO_4_ (CBP-01), induced ROS in murine sarcoma cells, which was associated with DNA cleavage, cell cycle arrest in G0/G1, and apoptosis of tumor cells (Polloni et al., 2019).

### 4.3 Antiangiogenic effect

Angiogenesis is typically initiated from existing blood capillaries and plays a critical role in providing oxygen and nutrients required for tumor growth, involving the activation of receptors such as VEGFR and dynamic interactions with the ECM (Tzeng and Huang, 2023; Lorenc et al., 2024). Tumor vessels are dysfunctional and anergic to inflammatory stimuli, providing an adverse TME, which favors cancer progression (Nowak-Sliwinska et al., 2023; Tzeng and Huang, 2023). A phenanthroline-linked Cu(II) complex, CPT8, showed antiangiogenic activity in HUVEC cell spheroids through inhibition of VEGF. The Cu(II) complex also presented cytotoxic and antimetastatic effects on invasive ovarian cancer cells, cleaving DNA and inhibiting MMP-2 (Shi et al., 2018).

### 4.4 Proteasome inhibition

Autophagy and the ubiquitin-proteasome system (UPS) are pathways that destroy old organelles or misfolded proteins. UPS consists of ubiquitin, ubiquitin-activating enzyme (E1), ubiquitin-conjugating enzyme (E2), ubiquitin ligase enzymes (E3), 26 S proteasome, deubiquitin enzyme (DUBs), and target proteins (Liu et al., 2025). This system is a potential therapeutic target, as transformed cells require high protein renewal. In addition to proteolysis, the UPS regulates DNA repair, stress response, and proliferation, favoring tumor progression (Liu et al., 2025; Wang et al., 2025a). Cu(II) complexes have also been reported to target the UPS, leading to the accumulation of ubiquitinated proteins and induction of apoptosis in cancer cells. (Li et al., 2019). A novel Cu(II) Schiff base complex demonstrated an antitumor effect mediated by UPS inhibition in 7,12-dimethylbenz [a]anthracene-induced cutaneous squamous cell carcinoma in female Swiss albino mice. The compound exhibited catalytic activity against the 20 S proteasome (El Yaagoubi et al., 2024).

### 4.5 Inhibition of telomerase

Finally, telomeres are TTAGGG repeats at the ends of the chromosome responsible for preserving genomic integrity, protecting chromosomes from unchecked degradation, and preventing aberrant activation of a DNA damage response. However, overexpression of the telomerase enzyme is a cancer hallmark, providing replicative immortality. Drugs targeting telomerase have also been investigated, showing limited effects on normal cells, as most somatic cells exhibit low telomerase activity (Baylie et al., 2025). A Cu(II) complex of dasatinib ([Cu(DAS + H) (NO_3_)_2_(H_2_O)]NO_3_·(H_2_O)·(CH_3_OH)) inhibited the activity of telomerase in hepatocarcinoma cells (Qin et al., 2018).

## 5 Cu complexes in the treatment of TNBC

The screening of metal complexes for the treatment of TNBC has advanced significantly in recent years. In contrast to classical metallopharmaceuticals, such as platinum analogues, the new metal-based complexes have undergone extensive structural modifications to reach selective activity against TNBC, overcoming resistance to conventional drugs (Muhammad et al., 2024). Although selectivity is a critical parameter, only a few studies include these data.

In the study by Karpagam and colleagues (2022), four Cu(II) complexes covalently linked with pyridyl ethyl amine conjugated to folic acid were evaluated in the TNBC cell line, MDA-MB-231 [Cu(FAA) (5,6-dmp) (H_2_O)]2^+^ was defined as the most promising and induced DNA damage through the reduction of Cu(II) to Cu(I) and its interaction with O_2_•−. Furthermore, the authors demonstrated that the complex was 200 times more cytotoxic to the TNBC cell line (IC_50_ = 0.54 µM) than to lymphocytes (IC_50_ = 108 µM), highlighting its selectivity (Karpagam et al., 2022).

In the TNBC line, HCC-1806, the Cu(I)-nicotinate complex (CNC) induced apoptosis, with overexpression of caspase 3, downregulation of survivin, and cell cycle arrest in G0/G1 (Abdel-Mohsen et al., 2019). Survivin is a multifunctional protein that induces proliferation and inhibits apoptosis, correlated to chemo- and radio-resistance (Albadari and Li, 2023). Fan and collaborators evaluated a Cu(II) complex associated with silicylate and phenanthroline (Cu(sal) (phen)) that significantly reduced tumor size in a Balb/c-nu model xenografted with MDA-MB-231 cells, with downregulation of Bcl-2 and survivin and no difference in weight between the treated groups and the control (Fan et al., 2017).

In 2024, Myeza treated MDA-MB-231 cells with a Cu 8-aminoquinoline-naphthyl complex (Cu8AqN) and observed an increase in the expression of caspases 3 and 7, which are apoptosis activators. Furthermore, the complex was three times more cytotoxic to the TNBC cell line (IC_50_ = 3.31 µM) than to the non-tumorigenic MCF 10A cell line (IC_50_ = 9.45 µM) (Myeza et al., 2024). In two other works, MDA-MB-231 cells treated with a dinuclear Cu(II)[(o-fen)Cu(H_2_O)_2_](NO_3_) and Cu(II) Complex [(o-phen)Cu(L)Cu(o-phen)](NO3) exhibited higher ROS production, DNA cleavage, and cell cycle arrest at the S and G2/M phases. The complexes also exhibited lower toxicity toward the non-tumorigenic MCF 10A cell line at higher concentrations (IC_50_ = 50 µM/75 μM, respectively) compared to the TNBC cell line (IC_50_ = 1.86 µM/0.41 µM, respectively) (Paliwal et al., 2024; Paliwal et al., 2025).

Chu and co-authors suggested, in their study, that the death mechanism mediated by Cu associated with disulfiram (DSF/Cu) in MDA-MB-231 cells was ferroptosis, since the compound increased intracellular iron levels, reduced glutathione expression, lipid peroxidation, and upregulated genes related to ferroptosis, especially Heme-Oxygenase-1 (*HMOX-1*) (Chu et al., 2023). Alajroush and colleagues treated MDA-MB-231 cells with [Cu(acetylethTSC)Cl]Cl·0,25C_2_H_5_OH complex and detected a significant increase in caspase-1 expression, suggesting cell death via pyroptosis. Despite the biological activity, the authors suggested that the compound was not selective when evaluated in the MCF 10A cell line (Alajroush et al., 2024).

Hou and colleagues noted, in Balb/c-nu model xenografted with MDA-MB-231 cells, that {[Cu_4_(C_13_H_17_O_3_NS)_4_]}_2_ inhibited Bcl-2 and increased the expression of caspase 9 and Bax. Simultaneously, the complex reduced the density of vascular endothelial cells, hindering angiogenesis (Hou et al., 2021). Suppression of angiogenesis by Cu complexes was also reported by Zhang and colleagues, which worked with two Cu(II) complexes, [Cu_4_(C_12_H_14_N_2_O_4_S)_4_] and [Cu_4_(C_12_H_13_NO_2_SClBr)_4_]_2_·4H_2_O, that reduced the expression of VEGFR2, Focal Adhesion Kinase (FAK), Serine/Threonine Kinase (AKT), and extracellular signal-regulated kinase 1 (ERK1) from MDA-MB-231 cells (Zhang P.-L. et al., 2020). Xu and colleagues evaluated the Cu complexes [Cu_4_(C_12_H_14_N_2_O_4_S)_4_] and [Cu_4_(C_12_H_13_NO_2_SClBr)_4_]_2_·4H_2_O in MDA-MB-231 cells and detected the downregulation of the proteins VEGFR2, FAK, AKT, and ERK1. Furthermore, in Balb/c-nu xenografts with MDA-MB-231 cells, the compound exhibited antitumor and anti-inflammatory effects, characterized by reduced expression of NF-κB, TNF-α, and IL-1β, and induced cell death via cuproptosis. No significant weight reduction was observed in the treated group compared to the control, nor were there alterations in the histopathological analyses of the heart, lung, spleen, and liver. Furthermore, it exhibited lower nephrotoxicity than cisplatin (Xu B.-B. et al., 2024). Cu(II) complex linked to phenanthroline and triphenylphosphonium (CPT8) also inhibited angiogenesis and metastasis in the TNBC model. CTP8 blocked vasculogenesis through downregulation of vascular endothelial cadherin, MMP-2, and MMP-9 in Balb/c-nu model xenografted with MDA-MB-231 cells, without significant toxicity (Zheng et al., 2023).

The Cu(II) complex synthesized with S-benzyldithiocarbazate and 3-acetylcoumarin [Cu(SBCM)2], in turn, reduced the metastatic phenotype of MDA-MB-231 TN cells by reducing the expression of the mutant *TP53* and MMP-2 protein, with lower cytotoxicity toward the HFF cell line (IC_50_ = 31.8 µM) compared to the TN cell line (IC_50_–18.7 µM) (Foo et al., 2018). Espindola-Moreno and colleagues evaluated the complex [Cu(HL) (NO_3_)]_2_·2H_2_O at low concentrations in MDA-MB-231 CD44+/CD24-cells. Treatment reduced the aggressive phenotype, decreasing the percentage of CD44+/CD24-cells from 98.6% to 68.3% after treatment for 24 h (Espindola-Moreno et al., 2025).

In the study by Li and colleagues (2019) five Cu(II) complexes [Cu(OH-PIP) (Phe)Cl], [Cu(OH-PIP) (Gly) (H2O)]NO_3_·2H_2_O [Cu(OH-PIP) (Ala) (Cl)]·H_2_O, [Cu(OH-PIP) (Met)]PF6·2H_2_O e [Cu(OH-PIP) (Gln) (H2O)](Cl)·3H_2_O downregulated Bcl-2 and caspase 3 and upregulated Bax and cleaved PARP in MDA-MB-231 and CAL-51 TNBC cells (Li et al., 2019). In 2023, Li together with other collaborators, carried out a study with results similar to those of 2019, but with five other Cu(II) complexes [Cu(OH-PIP) (Tyr) (H_2_O)]PF_6_, [Cu(m-OH-PIP) (Gly)]NO_3_·2,25 H_2_O, [Cu (Cl-PIP) (Gly) Br], [Cu (F-PIP) (Tyr) Cl] e [Cu (F-PIP) (Phe) Br]. The complexes modulated EMT in TNBC CAL-51 cells by increasing E-cadherin expression and decreasing Vimentin and Nanog. Furthermore, the complexes reduced the size of mammospheres, controlling triple-negative CSCs (Li et al., 2023).

TNBC cells, HCC 1806, treated with Cu(I)-(nicotinic acid)_2_Cl·2H_2_O (CNC), showed significantly reduced NOTCH1 and JAG1 expression, accompanied by increased autophagy (Abdel-Mohsen et al., 2024). Moreover, purinergic signaling can also be modulated in TNBC. MDA-MB-231 cells exhibit significant ectonucleotidase activity—enzymes that hydrolyze ATP, adenosine diphosphate (ADP), and adenosine monophosphate (AMP), thereby altering nucleotide levels, particularly adenosine, which is associated with an immunosuppressive TME (da Silva F. C. et al., 2022). A Cu(II) complex containing a long-chain aliphatic hydrazide and 1,10-phenanthroline ([Cu(dodh) (1,10-phen) (H_2_O)](NO_3_)_2_.H2O) modulated ectonucleotidase activity, leading to AMP accumulation, suggesting that Cu can alter adenosine levels in TNBC cells, MDA-MB-231. Furthermore, the compound demonstrated selectivity towards the non-tumorigenic MCF 10A cell line (Selective Index = 11.40) (Ferreira et al., 2024).

Interestingly, long non-coding RNAs (lncRNAs) are also involved in Cu metabolism and cancer aggressiveness. Rocha et al. (2025) demonstrated that, following a 48-h treatment of MDA-MB-231 cells with the complexes [Cu(clmp) (btacl) (NO_3_)] and [Cu(memp) (bta) (NO_3_)]·H_2_O, there was a significant reduction in the expression of the genes *lncATB*, *lncBANCR*, *lncBLACAT2*, *lncLINC00941*, and *lncOIP5-AS1*, which are associated with EMT, with significant selectivity (Selective Index of 2.40 and 3.78, respectively) relative to the non-tumorigenic MCF 10A cell line after 48 h of treatment (Rocha et al., 2025).

In the 1980s, a family of ternary Cu complexes was developed; in the early 1990s, these prototypes were patented as casiopeins. *In vitro*, [(4,4′-dimethyl-2,2′-bipyridine) (acetylacetonate)copper (II)]nitrate (CasIII-ia) was cytotoxic against MDA-MB-231 cells with Selective Index = 4.00 when compared to MCF 10A cells. *In vivo*, in a Balb/c-4T1 model, it significantly reduced tumor size (Aguilar-Jiménez et al., 2022). Casiopein II-gly, ([Cu(4,7-dimetil-1,10-fenantrolina) (glicinato)]NO_3_), in MDA-MB-231 and MDA-MB-468 cell lines, altered mitochondrial metabolism (Pacheco-Velázquez et al., 2018). Furthermore, Resendiz-Acevedo and collaborators also observed that CasII-gly impacts TNBC cell metabolism, including phospholipid biosynthesis and the metabolism of betaine, methionine, carbohydrates, and nucleotides. Thus, CasII-gly may control the metastatic process (Resendiz-Acevedo et al., 2021). González-Ballesteros and colleagues also evaluated CasIII-ia and CasII-gly *in vitro* and *in vivo*. Both inhibited the random and chemotactic migration of MDA-MB-231 cells and reduced tumor volume and weight in the Balb/c-4T1 model. Additionally, they significantly decreased metastatic foci in treated mice, with no significant difference in weight compared to the control group in animals monitored for 21 days (González-Ballesteros et al., 2024). Metabolically, the complexes altered phospholipid and phosphatidylcholine biosynthesis, the citric acid cycle, ketone body metabolism, and the mitochondrial electron transport chain (Resendiz-Acevedo et al., 2025).

Elesclomol is a copper compound that has also been showing promising results. It is a small molecule that acts as a Cu ionophore, promoting its intracellular accumulation. Tsvetkov and colleagues demonstrated that treating lung adenocarcinoma cell lines with low concentrations of elesclomol (40 nM) for just 2 h resulted in a 15- to 60-fold increase in intracellular Cu levels, inducing cell death. Importantly, the mechanism did not include apoptosis, ferroptosis, or necroptosis and, interestingly, was mediated by cuproptosis (Tsvetkov et al., 2022). Disulfiram, in turn, exhibits enhanced anticancer activity in the presence of Cu(II) ions. The effects of DSF/Cu(II) on breast and hepatocellular carcinoma cells include increased ROS production, inhibition of angiogenesis, and blockade of NF-κB activity (Kang et al., 2023; Chu et al., 2023).

Preclinical studies with Cu complexes are summarized in Supplementary Table S1. Overall, the results observed across the cited studies highlight the importance of coordination architecture, ligand nature, oxidation state, stability in biological media, and the cellular uptake of Cu complexes for effective antitumor activity. Different hallmarks, such as apoptosis, ferroptosis, pyroptosis, cuproptosis, autophagy, and angiogenesis, are described, and multiple pathways are evaluated, including Bcl-2/Bax, VEGFR2/AKT/ERK, NOTCH, EMT, inflammation, ROS, and purinergic signaling. The most robust studies are those that combine structural characterization of the complex, systematic comparison with free ligands or related compounds, independent cellular assays, mechanistic studies, and, whenever possible, validation in animal models. Redox state modulation is the most widely explored aspect, given that the Cu(I)/Cu(II) couple can act as a reversible redox system. Glutathione and other intracellular reducing agents can facilitate the reduction of Cu(II) to Cu(I), while oxygen can promote reoxidation, creating a cycle that increases oxidative stress. However, this does not constitute a proof of concept, but rather a dominant rationale.

Certain ligand families appear repeatedly, notably 1,10-phenanthrolines and aromatic diimines, Schiff bases, hydrazones, N-acylhydrazones, semicarbazones, and thiosemicarbazones, as well as ligands containing O-donor and N-donor groups and mixed N/O or N/S/O systems. Aromatic diimines appear to stabilize Cu(II) and provide an aromatic surface capable of interacting with biomolecules. Thus, minor structural changes in the peripheral groups can modify DNA interaction and cytotoxicity. Hydrazones and N-acylhydrazones constitute another particularly promising family, as they allow for the simultaneous control of the protonation state and the potential for dimerization. Thiosemicarbazones and thiocarbohydrazones, in turn, incorporate a sulfur atom with a high capacity for coordination with copper. Ligands containing N, S, and O exhibit great coordination versatility and can give rise to mononuclear or dinuclear complexes. However, the presence of sulfur can significantly alter the redox potential, overall charge, complex stability, and interaction with intracellular thiols. It is important to note that dimerization can increase the density of metal centers, generate new geometries, and favor interactions that would, in principle, enhance potency. Conversely, it can also increase molecular size, modify solubility, and alter internalization kinetics. Thus, the available results indicate that the structure-activity relationship (SAR) of the complexes is multidimensional. There is no simple relationship in which higher lipophilicity, greater stability, or higher DNA affinity always yields greater activity. On the contrary, potency appears to result from a balance among stability, lipophilicity, charge, geometry, redox capacity, accessibility of coordination sites, and intracellular distribution.

Furthermore, the available evidence supports the statement that some Cu complexes can achieve meaningful tumor cell selectivity, but this conclusion is based on a small number of direct comparisons. For most of the highly potent TNBC complexes, selectivity cannot currently be assessed because normal-cell IC_50_ values were not reported. Thus, an important limitation of the literature is that potency has been investigated much more systematically than safety. The fact is that the same redox properties that make Cu biologically important also make excess intracellular Cu potentially toxic. Consequently, successful clinical development will depend on controlling the localization and intracellular release of Cu, rather than simply maximizing Cu delivery.

Finally, studies available to date highlight the cytotoxicity of Cu complexes, though their effects on mechanisms of progression remain poorly understood. Metastatic activity, for instance, has been demonstrated in migration assays and through the expression analysis of known targets, yet lacks validation in more complex models. Nevertheless, there is a clear drive to develop complexes featuring sufficiently controlled redox reactivity, ligand architectures capable of directing specific molecular interactions, and pharmacokinetic properties that enable the selective targeting of tumor cells or compartments. This approach tends to yield greater therapeutic potency, improved selectivity, and, potentially, more consistent antimetastatic activity than strategies based solely on enhancing the reactivity of the metal center.

Most studies were carried out on MDA-MB-231 cells, which do not fully represent the molecular heterogeneity of TNBC. Long-term toxicity, pharmacokinetics, biodistribution, or interactions with the tumor microenvironment and immune system are not described. Therefore, although Cu-based complexes demonstrate substantial potential as multitarget therapeutic candidates for TNBC, further investigations employing patient-derived models, comprehensive omics approaches, and translational *in vivo* studies are necessary to establish their safety, specificity, and clinical applicability. In this context and considering the mechanisms related to Cu binding and transport, CTR1/SLC31A1, ATP7A/ATP7B, and the ATOX1–ATP7A/B axis warrant consideration. CTR1 (SLC31A1) controls Cu uptake and provides a relatively direct link between the tumor phenotype and Cu dependency, potentially identifying a TNBC subpopulation with higher Cu demand or mobilization. However, increased CTR1 expression could either enhance the uptake of the complex or the released Cu or facilitate greater homeostatic adaptation or therapeutic resistance, factors that require further investigation. Furthermore, it is necessary to demonstrate that CTR1 overexpression correlates with increased sensitivity to the specific complex, rather than merely reflecting intracellular exposure to the metal. ATP7A, in turn, is involved in the distribution, utilization, and export of Cu, as well as in supplying the metal to Cu-dependent proteins. Thus, inhibiting ATP7A could prevent the cell from properly utilizing or exporting Cu. Indeed, experimental evidence already indicates that interfering with intracellular Cu trafficking can be cytotoxic to TNBC (Karginova et al., 2019), highlighting the importance of the ATOX1–ATP7A/B axis and aggressive subtypes such as TNBC-BL.

The fact is that the clinical use of some synthetic and natural substances has been limited due to their poor absorption, distribution, metabolism, and excretion profiles. To address these limitations, several methodologies have been developed and implemented, especially those based on Cu and nanotechnology. Curcumin, for example, was complexed with Cu(II) and subsequently encapsulated into poly (styrene)-co-maleic acid micelles. The resulting nanoformulation exhibited dose-dependent cytotoxicity and significantly inhibited tumor growth Balb/c-4T1 model. Notably, the incorporation of Cu(II) increased the cytotoxic potential of curcumin, while its encapsulation improved stability, without evident side effects (Greish et al., 2018). Hu and colleagues also showed the advantages of nanoformulations containing Cu ions in TNBC cells. A poly [(2-(pyridin-2-yldisulfanyl)ethyl acrylate)-co-[poly (ethylene glycol)]] (PDA-PEG/copper) polymer containing Cu(II) (Poly-Cu) was designed and induced the death of 4T1 cells by inhibiting proteasome function. In the BALB/c-4T1 model, it was demonstrated that Poly-Cu combined with anti-PD-L1 exerts a synergistic effect and suppresses the progression of the TNBC model without systemic adverse effects (Hu et al., 2023).

Finally, only three Cu complexes, casiopeins, elesclamol, and disulfiram-copper (DSF/Cu(II)), are described in clinical trials (Krasnovskaya et al., 2020; Molinaro et al., 2020). After 30 years and promising preclinical results, CasIII-ia was selected for submission to the Mexican regulatory agency (COFEPRIS), marking the beginning of the first phase I clinical trial of a Cu complex in Mexico. The study is ongoing, and recent data have not yet been published (Aguilar-Jiménez et al., 2023). A phase III clinical trial of elesclomol in combination with paclitaxel was conducted in patients with advanced melanoma who had not received prior chemotherapy. Elesclomol combined with paclitaxel did not significantly improve progression-free survival (O'Day et al., 2013). Elesclomol was also combined with the AKT inhibitor MK2206, restoring sensitivity to cuprotosis and suppressing tumor growth in Balb/c-nu model xenografted with MDA-MB-231 cells (Sun et al., 2025). Finally, Phase I–III clinical trials evaluating DSF/Cu(II) for the treatment of metastatic breast and liver cancer are underway (Hangan et al., 2026). Updates on the study are unavailable, which limits conclusions regarding its clinical use.

Comparisons with therapies involving other metallodrugs are relevant. Platinum-based compounds (cisplatin, carboplatin, oxaliplatin) constitute the main clinically established class. They exhibit well-documented nephrotoxicity, ototoxicity, and neurotoxicity (Mishra et al., 2025). In platinum-refractory TN cells, Cu compounds have demonstrated cytotoxicity regardless of sensitivity status. In a panel of cancer cell lines with varying platinum sensitivity profiles, four tested copper complexes exhibited IC_50_ values ​​lower than those of cisplatin across all evaluated cell lines, whether sensitive or resistant (Wehbe et al., 2017). Indeed, Cu exhibits mechanisms of action, including ROS generation, DNA interaction, and the induction of cuproptosis, that are largely independent of the DNA platination pathway exploited by cisplatin (Silva et al., 2025).

Ruthenium complexes, in turn, occupy an intermediate position in this landscape. Several Ru(II)/Ru(III) compounds, such as BOLD-100 and RAPTA-C, have already advanced to clinical trials and preclinical studies, respectively, as anticancer agentes agents (D'Amato et al., 2023). Like Cu, they share the rationale of exploiting differences in redox potential and metal-binding properties between tumor and healthy cells. However, despite decades of preclinical development and the successful testing of some of these complexes in clinical trials, none have become commercially available to date. (Bhattacharya et al., 2025). However, Cu complexes remain at a comparatively earlier stage of development, particularly for the treatment of TNBC. The challenges of translation are intriguing. The primary barriers relate less to a lack of antitumor activity and more to the difficulty of transforming heterogeneous mechanistic results into a predictable, selective, and pharmacologically controllable therapeutic strategy. These key barriers are: (i) the absence of a validated predictive biomarker, (ii) the molecular heterogeneity of TNBC, (iii) uncertainty regarding the biologically active Cu species, (iv) the therapeutic window, (v) pharmacokinetics/biodistribution, and (vi) a scarcity of clinical validation and robust metastatic models. Cu complexes are promising. The rational design of ligands makes it possible to exploit the unique properties of Cu complexes by modulating various pathways (Figure 5). Consequently, the versatility of their mechanisms of action, combined with novel nanotechnological approaches, offers new opportunities to overcome drug resistance, enhance selectivity, and minimize adverse effects.

**FIGURE 5:**
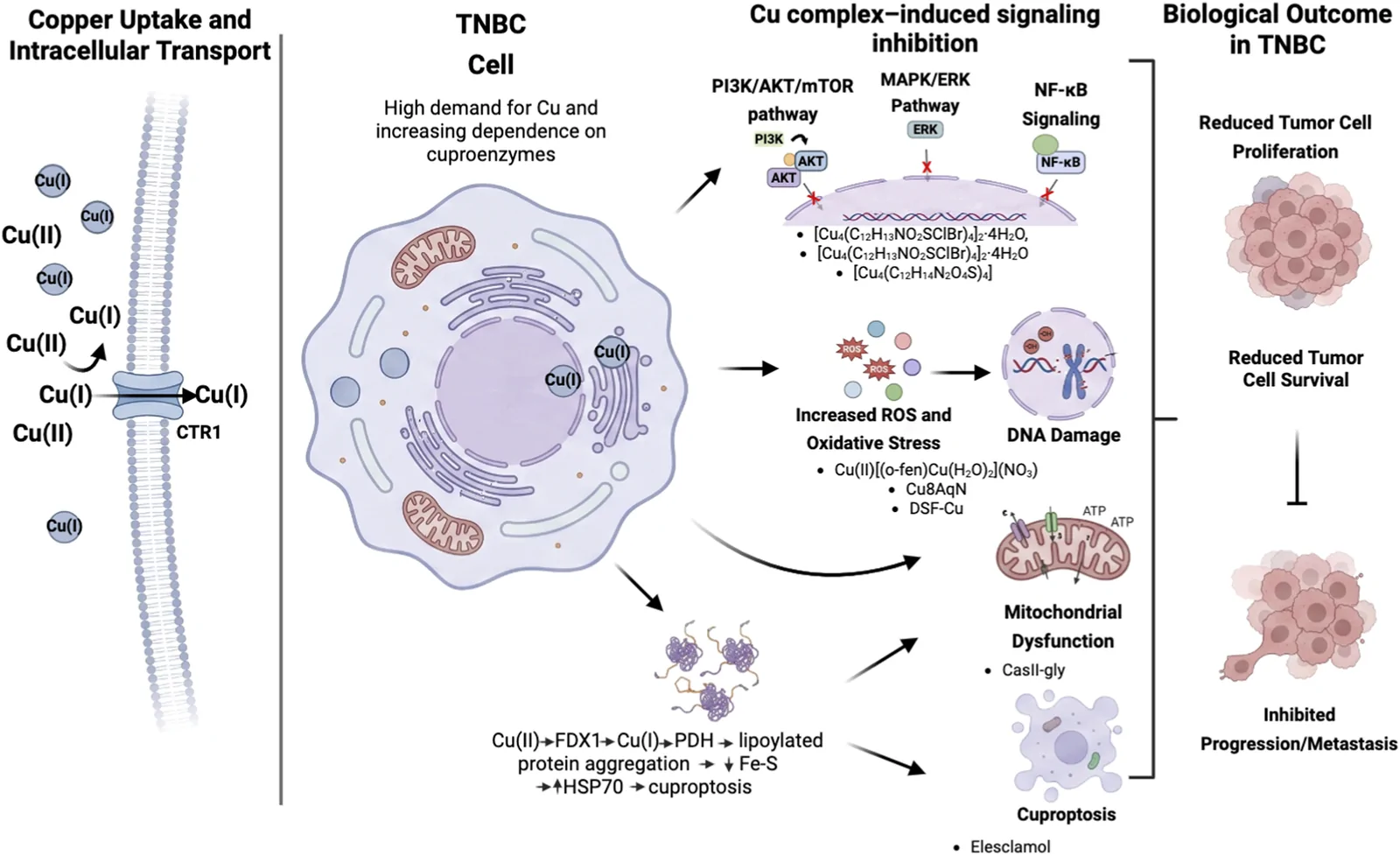
Copper biology and anticancer mechanisms in triple-negative breast cancer (TNBC). Cu(II) is reduced to Cu(I) and internalized by TNBC cells via the high-affinity copper transporter 1 (CTR1). In TNBC cells, Cu(I) fuels three convergent mechanisms. First, inhibition of oncogenic signaling: representative complexes ([Cu_4_(C_12_H_14_N_2_O_4_S)_4_], [Cu_4_(C_12_H_13_NO_2_SClBr)_4_]_2_·4H_2_O) downregulate the PI3K/AKT/mTOR, MAPK/ERK, and NF-κB pathways, reducing proliferative and inflammatory signaling. Second, the generation of reactive oxygen species (ROS) leads to oxidative stress and DNA damage, exemplified by Cu8AqN, disulfiram/copper (DSF/Cu), and the dinuclear complex Cu(II) (o-fen)Cu(H_2_O)_2_. Third, cuproptosis is triggered when Cu(II) is reduced by ferredoxin 1 (FDX1) to Cu(I), promoting lipoylation of pyruvate dehydrogenase complex (PDH) subunits, proteotoxic protein aggregation, loss of iron-sulfur (Fe-S) cluster-containing proteins, and accumulation of heat shock protein 70 (HSP70), collectively driving mitochondrial dysfunction and cuproptotic cell death: illustrated by elesclomol. Casiopein II-glycinate (CasII-gly) altered mitochondrial metabolism. Together, these mechanisms converge on reduced tumor cell proliferation, survival, and metastatic progression in TNBC. Representative complexes and compounds discussed in the text are indicated within each panel. CTR1: copper transporter 1; PI3K: phosphoinositide 3-kinase; AKT: protein kinase B; mTOR: mechanistic target of rapamycin; MAPK: mitogen-activated protein kinase; ERK: extracellular signal-regulated kinase; NF-κB: nuclear factor kappa B; ROS: reactive oxygen species; DSF/Cu: disulfiram-copper complex; FDX1: ferredoxin 1; PDH: pyruvate dehydrogenase complex; Fe-S: iron-sulfur; HSP70: heat shock protein 70; CasII-gly: casiopein II-glycinate; TNBC: triple-negative breast cancer. The figure was created with BioRender.com. Barbosa, L. (2026). https://BioRender.com/7zp9a2f.

## 6 Concluding remarks

TNBC is a BC subtype with high intrinsic and extrinsic heterogeneity. Despite increasing efforts to elucidate its molecular mechanisms, effective and more selective therapeutic strategies remain limited. In this scenario, cytotoxic chemotherapy is the standard treatment approach. New drugs have been emerging to overcome these challenges, with Cu complexes deserving attention. In fact, the participation of Cu in oxidation-reduction reactions has a significant impact on cell metabolism, survival, and growth. Furthermore, it combines biocompatibility and bioavailability. Cu complexes have been designed and evaluated in TNBC cells, modulating oxidative stress, cell death, and angiogenesis. The number of complexes tested is increasing, but few have progressed to clinical trials. Thus, biomarker validation and patient stratification, combined with rational design and nanotechnology, are strategies that need to be explored, thereby expanding the possibilities for treating patients with TNBC.

## In memoriam

This work is dedicated to Luiz Ricardo Goulart Filho (in memoriam), PhD, a brilliant Brazilian scientist who deceased due to COVID-19.
