# Supplementary material for: Copper complexes in the triple-negative breast cancer landscape: advances and opportunities
Source: Front Mol Biosci. 2026 Oct 12;13:1931263. doi: 10.3389/fmolb.2026.1931263 (PMC13696109; doi:10.3389/fmolb.2026.1931263)
Supplement: Supplementary file 1 [file Table1.docx]

Supplementary Material

**Supplementary Table 1.** Copper complexes with promising effects on triple-negative breast cancer (TNBC) models.

| **Copper complex** | **TNBC model** | **Mechanism of action** | **Potential targets** | **Reference** |
| --- | --- | --- | --- | --- |
| Cu(sal)(phen) | MDA-MB-231 cells  BT-20 cells  Balb/c-nu model xenografted with MDA-MB-231 cells | Apoptosis  Inhibition of tumor growth in vivo | ↓Bcl-2  ↓Bcl-xL  ↓Ki67  ↓Survivin  ↑Cleaved PARP | (Fan et al., 2017) |
| [Cu(SBCM)_2_] | MDA-MB-231 cells | Apoptosis  Cell cycle arrest in G2/M phase  Metastasis inhibition | ↓Mutate p53  ↓ MMP2 | (Foo et al., 2018) |
| [Cu(OH-PIP)(Phe)Cl],  [Cu(OH-PIP)(Gly)(H_2_O)]NO_3_·2H_2_O,  [Cu(OH-PIP)(Ala)(Cl)]·H_2_O,  [Cu(OH-PIP)(Met)]PF6·2H_2_O and  [Cu(OH-PIP) )(Gln)(H_2_O)](Cl)·3H_2_O | MDA-MB-231cells  CAL-51 cells | Antiproliferative effect  Apoptosis  CSC inhibition | ↓Subunidade β5 catálitica proteasomal 20S  ↑Bax  ↓Bcl-2  ↓Caspase 3  ↓ ALDH  ↓CD44 | (Li et al., 2019) |
| CNC | HCC-1806 cells | Apoptosis and necrosis  Cycle arrest in G0  Antioxidant effect  Cleavage of plasmid DNA | ↑Caspase 3  ↑ SOD  ↓Survivin | (Abdel-Mohsen et al., 2019;  Karpagam et al., 2022) |
| [Cu_4_(C_12_H_14_N_2_O_4_S)_4_] and  [Cu_4_(C_12_H_13_NO_2_SClBr)_4_]_2_·4H_2_O | MDA-MB-231 cells | Antiproliferative effect  Apoptosis  Antimetastatic  Antiangiogenic | ↓VEGFR2  ↓FAK  ↓AKT  ↓Erk1 | (Zhang et al., 2020b) |
| {[Cu_4_(C_13_H_17_O_3_NS)_4_]}_2_ and  [Cu_4_(C_20_H_31_O_2_NS)_4_] | Balb/c-nu model xenografted with MDA-MB-231 cells | Apoptosis  Antiangiogenic | ↓Bcl-2  ↑Bax  ↑Caspase 9 | (Hou et al., 2021) |
| [Cu(OH-PIP)(Tyr)(H_2_O)]PF6,  [Cu(m-OH-PIP)(Gly)]NO_3_2.25H_2_O,  [Cu(Cl-PIP)(Gly)Br,  [Cu(F-PIP)(Tyr)Cl] and  [Cu(F-PIP)(Phe)Br] | CAL-51 cells  MDA-MB-231 cells | Antiproliferative effect  Apoptosis  EMT inhibition | ↓Bcl-2  ↓pro-PARP  ↓Nanog  ↓Vimentin  ↓Caspase 3  ↑Bax  ↑E-cadherin | (Li et al., 2023) |
| CPT8 | MDA-MB-231 cells | Antiproliferative effect  Apoptosis  Mitophagy  Antimetastatic  Antiangiogenic | ↑PINK1/Parkin  ↓ Nrf2  ↓VEGF  ↓CD34  ↓VE-cadherin  ↓MMP2/ MMP9  ↓Ki67 | (Zheng et al., 2023) |
| [Cu(dodh)(1,10-phen)(H_2_O)](NO_3_)_2_.H_2_O) | MDA-MB-231 cells | Clonogenicity inhibition  Upregulation of ecto-nucleoside triphosphate diphosphohydrolase 1 | ↑ AMP  ↓ ADO | (Ferreira et al., 2024) |
| (Cu8AqN) | MDA-MB-231 cells | Apoptosis  Oxidative stress | ↑Caspase 3  ↑Caspase 7 | (Myeza et al., 2024) |
| Cu(II)[(o-fen)Cu(H_2_O)_2_](NO_3_) | MDA-MB-231 cells | Oxidative stress  Cell cycle arrest in S/G2/M phase  Cleavage of DNA | ↑Caspase 3  ↑Caspase 7 | (Paliwal et al., 2024; Paliwal et al., 2025) |
| Cu(I)-(ácido nicotínico)_2_Cl-2H_2_O | HCC cells | Autophagy | ↓ NOTCH  ↓ HES1  ↓ JAG1 | (Abdel Mohsen et al., 2024) |
| [Cu(acetylethTSC)Cl]Cl·0,25C_2_H_5_OH | MDA-MB-231 cells | Piroptosis | ↑ROS  ↑Caspase 3  ↑Caspase 7  ↑Caspase 9  ↑Caspase 1 | (Alajroush et al., 2024) |
| **[**Cu(clmp)(btacl)(NO₃)] and [Cu(memp)(bta)(NO₃)]·H₂O, | MDA-MB-231 cells | EMT inhibition | ↓ *lncATB*  ↓ *lncBANCR*  ↓*lncBLACAT2*  ↓*lncLINC00941*  ↓*lncOIP5-AS1* | (Rocha et al., 2025) |
| Cu(C_12_H_8_N_2_)(C_12_H_13_O_2_N_4_S)_2_]·CH_3_CH_2_OH·_5_H_2_O | MDA-MB-231  Balb/c-nu tumor bearing MDA-MB-231 cells | Antiproliferative  Antiangiogenic  Inhibition of tumor growth in vivo  Cuproptosis  Anti-inflammatory | ↓VRGFR2  ↓FAK  ↓AKT  ↓Erk1  ↓NF-κB,  ↓TNF-α  ↓IL-1β  ↑FDX1  ↑HSP70 | (Xu et al., 2024a) |
| [Cu(**HL)**(NO₃)]_₂_·2H₂O | MDA-MB-231 cells | Antiproliferative | ↓CD44+/CD24- | (Espindola-Moreno et al., 2025) |
| CasII-gly | MDA-MB-231 cells  MDA-MB-468 cells | Affected mitochondrial metabolism  Disturbance of oxidative phosphorylation  Antiproliferative effect  Antimetastatic | ↓Vimentin  ↑E-cadherin | (Pacheco-Velázquez et al., 2018; Resendiz-Acevedo et al., 2021) |
| CasIII-ia | MDA-MB-231 cells  Balb/c-4T1 | Antiproliferative effect  Inhibition of tumor growth in vivo |  | (Aguilar-Jiménez et al., 2022b) |
| DSF/Cu | MDA-MB-231 cells  CHC cells | Ferroptosis  Oxidative stress  Lipid peroxidation | ↓ GPX4  ↓ GSH  ↑ HMOX1 | (Chu et al., 2023) |

**Abbreviations**: Apoptosis regulator Bcl-2 isoform beta (Bcl-2); BCL2-like 1 (Bcl-xL); KI67 Antigen, partial (Ki67); poli [ADP-ribose] polimerase 1 (PARP); matrix metallopeptidase 2 (MMP2); BCL2 associated X, apoptosis regulator (Bax); Aldehyde dehydrogenase (ALDH); CD44 molecule (CD44); Cu/Zn superoxide dismutase (SOD); soluble vascular endothelial growth factor receptor-2 (VEGFR2); focal adhesion kinase (FAK); serine/threonine kinase (AKT); extracellular signal- regulated kinase 1 (Erk1); TEN induced kinase 1 / parkin RBR E3 ubiquitina proteína ligase (PINK1/Parkin); Nuclear factor erythroid 2-related factor 2 (Nrf2); vascular endothelial growth factor (VEGF); CD44 molecule (CD34); matrix metallopeptidase 9 (MMP9); glutathione peroxidase 4 (GPX4); glutathione reductase (GSH); heme oxygenase 1 (HMOX1); Notch receptor 1 (NOTCH); hes family bHLH transcription factor 1 (HES1); jagged canonical Notch ligand 1 (JAG1); adenosine monophosphate (AMP); adenosine (ADO); Long noncoding RNA activated by TGF-β (lncATB), Bladder cancer-associated transcript 2 (lncBLACAT2), Long intergenic nonprotein coding RNA 941 (lncLINC00941), OIP5 antisense RNA 1 (lncOIP5-AS1); tumor necrosis factor (TNF-α); interleukin 1 beta (IL-1β); ferredoxin 1 (FDX1); heat shock protein 70 (HSP70). Epithelial-mesenchimal transition (EMT).
